# Performance Improvement of Pure Pursuit Algorithm via Online Slip Estimation for Off-Road Tracked Vehicle

**DOI:** 10.3390/s25144242

**Published:** 2025-07-08

**Authors:** Çağıl Çiloğlu, Emir Kutluay

**Affiliations:** Graduate School of Science and Engineering, Hacettepe University, Beytepe, 06800 Ankara, Türkiye; kutluay@hacettepe.edu.tr

**Keywords:** tracked mobile robots, path tracking, slip estimation, extended Kalman filter

## Abstract

The motion control of a tracked mobile robot remains an important capability for autonomous navigation. Kinematic path-tracking algorithms are commonly used in mobile robotics due to their ease of implementation and real-time computational cost advantage. This paper integrates an extended Kalman filter (EKF) into a common kinematic controller for path-tracking performance improvement. The extended Kalman filter estimates the instantaneous center of rotation (ICR) of tracks using the sensor readings of GPS and IMU. These ICR estimations are then given as input to the motion control algorithm to generate the track velocity demands. The platform to be controlled is a heavyweight off-road tracked vehicle, which necessitates the investigation of slip values. A high-fidelity simulation model, which is verified with field tests, is used as the plant in the path-tracking simulations. The performance of the filter and the algorithm is also demonstrated in field tests on a stabilized road. The field results show that the proposed estimation increases the path-tracking accuracy significantly (about 44%) compared to the classical pure pursuit.

## 1. Introduction

Advances in computing, sensors and data technologies are driving a growing interest and an increase in investments in unmanned ground vehicles (UGVs). These UGVs can be classified as tracked, wheeled or legged according to their actuation type. Off-road capability for most of these platforms is essential to negotiate unstructured and rough terrain. UGVs are utilized for disaster response, surveillance, mining, military and agriculture applications [1].

Tracked vehicles offer several advantages compared to their alternatives, depending on their operational use. These advantages include soft soil performance, terrain adaptability, enhanced traction and higher maneuverability in restricted spaces. The complex track–ground interaction makes the motion control problem of such vehicles a significant research area.

Kinematic controllers are widely used in motion control problems due to their simplicity, ease of implementation, robustness to varying parameters and low real-time computational demand on the processors. On the other hand, they lack accuracy in highly dynamic situations when friction forces, inertia or slip values become non-negligible. There have been several kinematic control strategies regarding the path-tracking problem of mobile robots. Among the abundant research, three concepts have become mainstream over the years: PID controllers, Stanley controllers and pure pursuit controllers.

Sabiha et al. [2] use a backstepping kinematic PID controller in combination with a sliding mode controller for the path tracking of a tracked vehicle. The gains are optimally calculated in real time, and the system is shown to be Lyapunov stable.

Pure pursuit and Stanley algorithms are among the most famous kinematic controllers due to their simplicity and general success over the years. In a study by Wang et al. [3], the Stanley algorithm performance is improved by a multi population genetic algorithm for an agricultural tractor. The research demonstrates that both path-tracking and stability characteristics can be improved. A parallel PID–Stanley algorithm is used by Fu [4] on a four-wheel steering mobile robot platform. By combining the lateral and longitudinal control problems, the path-tracking errors are decreased. The implementation of Stanley on an unmanned tracked platform is explored in a study by Subari [5]. Only straight-line following results are presented in a simulation domain for low-velocity operation (3 m/s).

The pure pursuit algorithm was first used in a mobile robot at Carnegie Mellon Institute of Robotics [6]. It was implemented on a skid steer wheeled robot. In the following years, it gained widespread popularity both in automotive and mobile robotics [7,8,9]. Since it is a kinematic controller, it does not require track–terrain friction parameters, which are significantly variable for an off-road vehicle and, therefore, difficult to know or calculate in real time. Thanks to its look-ahead nature, it starts to regulate control inputs before entering a curvature, which gives it a significant advantage compared to classical PID-type controllers. It requires very little field tuning. Morales et al. [10] use 2D laser scanners, along with the pure pursuit algorithm, to track both explicit and implicit paths. The results are verified on a tracked robot called Auriga-α. Wang et al. [11] apply pure pursuit to a tracked paver with an improved look-ahead distance formulation rather than being constant. The paper includes implementation on an actual vehicle with decreased lateral errors. The derivation of track velocities is the same as a differential drive robot (DDR) with no slip assumption. Operation velocities in the paper are less than 5 km/h because of the nature of the platform. In a more recent paper by Wu [12], a multi-sensor fusion algorithm is tested on a type of tracked construction machinery to track a path in weak GPS regions. An agricultural platform implementation of the pure pursuit algorithm is available in [13], where a variable look-ahead distance formulation is proposed. All the previously mentioned path-tracking studies [10,11,12,13] disregard slip and present results for low-velocity operation (< 5km/h).

The estimation of slip parameters for mobile robots is also an active research area. To avoid modeling the complex track/terrain interaction as the robot negotiates unstructured terrain, the robot’s instantaneous center of rotation (ICR) location is estimated. In an attempt to decrease the real-time computational cost, Martinez et al. [14] lay the groundwork for estimating the individual ICRs of right and left tracks by means of a high-fidelity dynamic model. They also perform test runs on an instrumented tracked robot, and using genetic algorithms as an optimization method, they extract the ICR locations. Both the simulation and genetic algorithm results are presented. An extended Kalman filter is used by Pentzer et al. [15] to estimate the locations of ICRs in real time as the vehicle travels along a route. The goal of the study is to perform dead-reckoning navigation in the absence of GNSS data. Non-linear kinematic state equations are implemented in the filter. The converging characteristics and ICR location estimation performance of the filter are demonstrated for a differential drive robot and a tracked robot. The histograms of ICR locations as robots negotiate a 1 km road are found to be clustered around the estimations predicted by the filter. This filter is modified by Zhao [16] by relating the ICR locations to centrifugal acceleration and road curvature. A total of six slip parameters are estimated in real time in combination with a model predictive controller (MPC). The extended Kalman filter implementation is verified by field tests on a 13.6-ton remote-controlled tracked vehicle. Another MPC scheme is presented in Liang et al. [17], where a robust MPC is used in conjunction with non-parallel distributed compensation and a polytopic model. The results are presented both in a co-simulation domain and in actual field tests. To decrease the steering effort of the driver, a robust state feedback controller with a parallel distribution method is proposed by Liang et al. [18]. Hardware in the loop tests in a co-simulation framework are conducted to assess the validity of the architecture.

Published motion control studies for heavyweight off-road vehicles with slip compensation are very limited in the literature. Furthermore, the application of pure pursuit controllers to heavyweight off-road tracked vehicles necessitates the investigation of slip values, which are disregarded in the existing pure pursuit literature. To fill this gap, we propose a two-stage controller. First, an extended Kalman filter (EKF), which was originally designed to perform dead-reckoning navigation, is introduced. This filter estimates the ICR locations of the right/left tracks in real time. Outputs of these estimations are then used to compute a variable artificial track width in order to regulate the speed differences between the inner and outer sprockets. The application of ICR estimates for a more accurate pure pursuit performance path tracking is the original contribution of the study. The proposed architecture’s performance enhancement is validated both in high-fidelity simulations and real-world tests. The proposed approach allows improved slip compensation compared to classical pure pursuit while keeping most of its inherent advantages, such as ease of implementation, robustness to varying terrain parameters and minimal on-field tuning. The results demonstrate that significant improvements can be achieved when this ‘slip-aware’ pure pursuit is utilized. A comparison of the proposed method with the existing literature is shown in Table 1.

The paper is organized as follows: Section 2 presents the formulation of the extended Kalman filter to estimate the ICR locations in real time. Section 3 introduces the high-fidelity dynamic model of the off-road vehicle used in simulations. Classical pure pursuit, EKF-enhanced pure pursuit controllers and their differences in velocity requests are explained in Section 4. The effectiveness of the proposed algorithm is investigated in a multi-body simulation environment in Section 5. The field results for the designed EKF and path-tracking performance comparisons are presented in Section 6. Section 7 presents a discussion about the conclusions of the paper, along with possible future works.

## 2. Extended Kalman Filter Design

**Figure 1:**
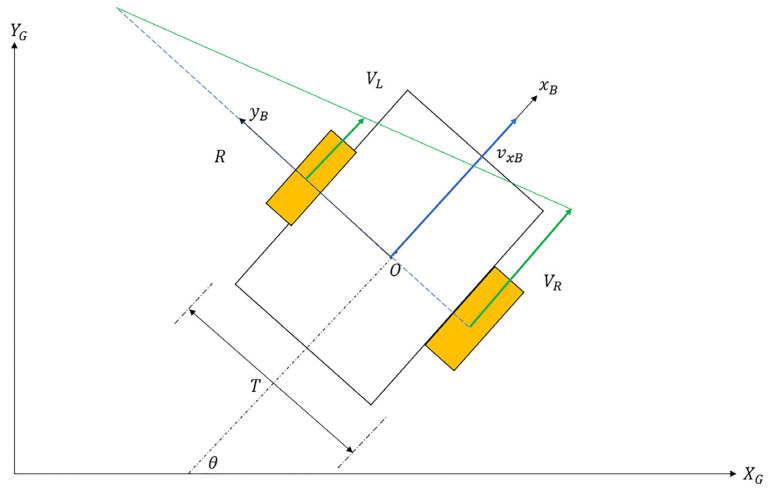
DDR kinematics.

**Figure 2:**
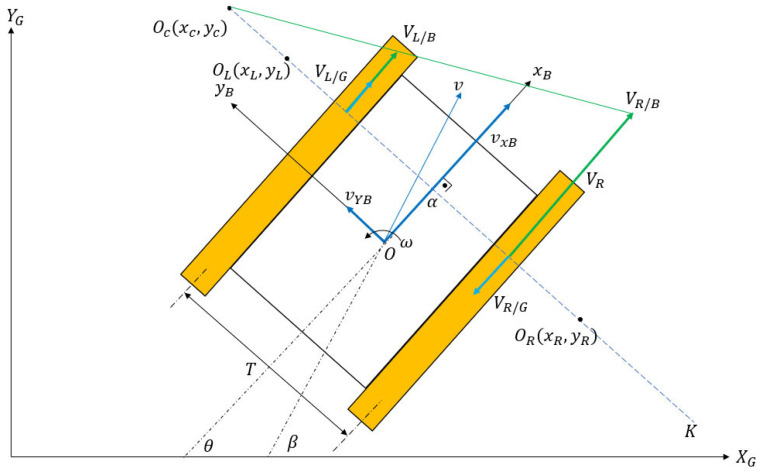
Tracked vehicle kinematics.

As a tracked vehicle executes a turn, the instantaneous centers of rotation (ICRs) for the right and left tracks become different than the vehicle’s overall center of rotation. The reason for this discrepancy is the existence of slip (both in lateral and longitudinal directions). Schematics of a differential drive robot (DDR) with no slip and a tracked vehicle performing the same maneuver are shown in Figure 1 and Figure 2, respectively.

**Figure 3:**
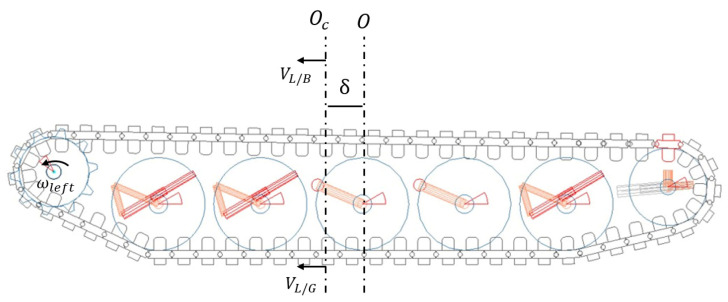
Side view of vehicle.

In Figure 2, $O_{c}$ is the vehicle’s body rotation center, and $O_{L}$ and $O_{R}$ are the individual rotation centers of right and left tracks. Their coordinates ($x_{L},y_{L},x_{R},y_{R}$) are specified in the body frame with respect topoint O. A tracked skid steer vehicle maneuvers by adjusting the angular speeds of the inner and outer tracks. In a typical maneuver, the outer track has a higher angular velocity than the inner track. The right track velocity with respect to the ground is denoted as $V_{R/G}$, while the right track velocity with respect to the body is denoted as $V_{R/B}$. Similarly, the left track velocities are denoted as $V_{L/G}$ and $V_{L/B}$. The inertial coordinate system is specified as $X_{G}$ and $Y_{G}$, and the body frame coordinate system is denoted by $x_{B}$ and $y_{B}$. T is the vehicle track width, which is the center-to-center distance between tracks in the lateral direction. The yaw rate of the vehicle is denoted as ω. Finally, $v_{xB}$ and $v_{yB}$ are the forward and lateral velocity components of the vehicle in the body frame. The following equations can be written by referring to Figure 2 and Figure 3:

(1) $$V_{R/G}=V_{R/B}-\omega_{R}*r_{spr}$$

(2) $$V_{L/G}=V_{L/B}-\omega_{L}*r_{spr}$$

(3) $$V_{R/B}=v_{xB}+\frac{T}{2}\omega$$

(4) $$V_{L/B}=v_{xB}-\frac{T}{2}\omega$$

(5) $$V_{R/G}=\omega \left(y_{R}-\left(-\frac{T}{2}\right)\right)$$

(6) $$V_{L/G}=\omega \left(y_{L}-\frac{T}{2}\right)$$

In the above equations, $\omega_{R}$ and $\omega_{L}$ are the sprocket angular velocities, and the sprocket pitch radius is depicted as $r_{spr}$. Combining Equations (1)–(6) and solving for ICR locations yields

(7) $$y_{R}=\frac{v_{xB}-\omega_{R}*r_{spr}}{\omega }$$

(8) $$y_{L}=\frac{v_{xB}-\omega_{L}*r_{spr}}{\omega }$$

The difference between the $V_{L/G}$ and $V_{L/B}$ vectors and their relations is shown in Figure 3.

The ICR location of the entire vehicle can be calculated by simple velocity relations:

(9) $$y_{c}=\frac{v_{xB}}{\omega }$$

(10) $$x_{L}=x_{R}=x_{c}=-\frac{v_{yB}}{\omega }$$

As specified by Pentzer [15], the ICR coordinates $y_{L}$,$\;y_{R}$ and $x_{L}$ remain bounded and finite because the numerators and denominators in Equations (7), (8) and (10) are infinitesimals of the same order. This statement is not valid for Equation (9).

For a given angular velocity of the right and left sprockets, the resulting body frame velocities in the presence of slip can be calculated using Equations (11)–(13):

(11) $$v_{xB}=\frac{\omega_{R}r_{spr}y_{L}-\omega_{L}r_{spr}y_{R}}{y_{L}-y_{R}}$$

(12) $$v_{yB}=\frac{\left(\omega_{L}r_{spr}-\omega_{R}r_{spr}\right)*x_{L}}{y_{L}-y_{R}}$$

(13) $$\omega =-\frac{\left(\omega_{L}r_{spr}-\omega_{R}r_{spr}\right)}{y_{L}-y_{R}}$$

The global frame velocities can be calculated using Equations (14) and (15):

(14) $${\dot{X}}_{G}=v_{xB}\cos\left(\theta \right)-v_{yB}\sin\left(\theta \right)$$

(15) $${\dot{Y}}_{G}=v_{xB}\sin\left(\theta \right)+v_{yB}\cos\left(\theta \right)$$

The state–space form of the combined equations is written in Equation (16), where vehicle states x are the global coordinates of the vehicle, yaw angle and ICR locations, with respect to point O in the body frame, respectively.

(16) $$\dot{x}=\left[\begin{array}{c} {\dot{X}}_{G}\\ {\dot{Y}}_{G}\\ \dot{\theta }\\ {\dot{y}}_{R}\\ {\dot{y}}_{L}\\ {\dot{x}}_{c} \end{array}\right]=\left[\begin{array}{c} v_{xB}\cos\left(\theta \right)-v_{yB}\;sin\left(\theta \right)+{PN}_{1}\\ v_{xB}\sin\left(\theta \right)+v_{yB}\;cos\left(\theta \right)+{PN}_{2}\\ -\frac{\left(\omega_{L}r_{spr}-\omega_{R}r_{spr}\right)}{y_{L}-y_{R}}+{PN}_{3}\\ {PN}_{4}\\ {PN}_{5}\\ {PN}_{6} \end{array}\right]\;$$

In Equation (16), PN_1_ to PN_6_ represent additive zero-mean Gaussian process noises. Similar to the justification presented in Martinez [14] and Pentzer [15], the ICR locations are assumed to remain bounded within a small region, but they do not necessarily lie on the tracks. They are treated as constant with random process noise. The validity of this assumption was further investigated in field tests. The state space equation was discretized by the Euler method, as shown in Equation (17):

(17) $$x_{k}=\left[\begin{array}{c} X_{Gk}\\ Y_{Gk}\\ \theta_{k}\\ y_{Rk}\\ y_{Lk}\\ x_{ck} \end{array}\right]=\left[\begin{array}{c} {X_{G\left(k-1\right)}+(v}_{xB\left(k-1\right)}\cos\left(\theta_{k-1}\right)-v_{yB\left(k-1\right)}\;sin\left(\theta_{k-1}\right))∆t+{PN}_{1}∆t\\ {Y_{G\left(k-1\right)}+(v}_{xB\left(k-1\right)}\sin\left(\theta_{k-1}\right)+v_{yB\left(k-1\right)}\;cos\left(\theta_{k-1}\right))∆t+{PN}_{2}∆t\\ \theta_{k-1}+\left(-\frac{\left(\omega_{L\left(k-1\right)}r_{spr}-\omega_{R\left(k-1\right)}r_{spr}\right)}{y_{L\left(k-1\right)}-y_{R\left(k-1\right)}}\right)∆t+{PN}_{3}∆t\\ y_{Rk-1}+{PN}_{4}∆t\\ y_{Lk-1}{+PN}_{5}∆t\\ x_{ck-1}+{PN}_{6}∆t \end{array}\right]$$

where

(18) $$v_{xB\left(k-1\right)}=\frac{\omega_{R\left(k-1\right)}r_{spr}y_{L\left(k-1\right)}-\omega_{L\left(k-1\right)}r_{spr}y_{R\left(k-1\right)}}{y_{L\left(k-1\right)}-y_{R\left(k-1\right)}}$$

(19) $$v_{yB\left(k-1\right)}=\frac{\left(\omega_{L\left(k-1\right)}r_{spr}-\omega_{R\left(k-1\right)}r_{spr}\right)*x_{L\left(k-1\right)}}{y_{L\left(k-1\right)}-y_{R\left(k-1\right)}}$$

The problem can be compactly written in the extended Kalman filter nomenclature, as shown in Equation (20):

(20) $$x_{k}^{-}=f\left(x_{k-1}^{+},u_{k-1},0\right)$$

The state vector $x_{k}^{-}$ is the a priori estimate at the current time step, $x_{k-1}^{+}$ is the a posteriori estimate from the previous time step, and $u_{k-1}$ is the vector of the sprocket velocity input from the previous time step, as shown in Equation (21). The third entry being zero means the process noise is set to zero for propagation:

(21) $$u_{k-1}=\left[\begin{array}{c} \omega_{L\left(k-1\right)}r_{spr}\\ \omega_{R\left(k-1\right)}r_{spr} \end{array}\right]$$

Once the inner parameters of the matrices are determined, the problem becomes a standard extended Kalman filter problem. Using the onboard GPS and IMU data, the measurement equations are shown in Equation (22), where MN_1_ to MN_3_ represent additive measurement noise:

(22) $$h_{k}=\left[\begin{array}{c} X_{Gk}+{MN}_{1}\\ Y_{Gk}+{MN}_{2}\\ \theta_{k}+{MN}_{3} \end{array}\right]$$

Jacobian evaluations, as well as equations for the propagation of the states, follow the same procedure outlined by Pentzer [15]. The detailed equations can also be found in Appendix A for the interested reader. From a high-level point of view, the extended Kalman filter outputs state estimations for the kth time instant using the input sprocket velocities and measured vehicle pose at the k-1th time instant. Table 2 summarizes all the parameters detailed in this section.

## 3. High-Fidelity Dynamic Model

To model the tracked vehicle, the multi-body software package MSC Adams (Hexagon AB, Stockholm, Sweden) 2022.1 version was used. The Adams Tracked Vehicle (ATV) module, which is an add-on to the original software, was utilized for modeling the physical components of the suspension. The physical modeling aspect allows all non-linearities, such as suspension travel, dampers and friction parameters between the track and ground, to be considered.

Shear stress ($\tau_{shear}$) along the track was calculated according to the formula proposed by Wong [19] and MSC Adams Product Documentation [20] (Equation (23)):

(23) $$\tau_{shear}=\left(c+Ptan\emptyset \right)\left(1-e^{-\frac{j}{K}}\right)$$

where c is the maximum shear stress at zero ground pressure (cohesion factor), ∅ is the internal shearing resistance angle, P is the ground pressure at the contact patch, j is the shear displacement of the track pad at the current time step, and K is the shear modulus of the terrain. Shear displacement is the integral of track shoe velocities with respect to the ground and was calculated at each time step (Equation (24)):

(24) $$j_{n}=j_{n-1}+dj$$

Shear displacement from the previous time step to the current time is denoted as dj. The contact force ($F_{shear}$) was calculated by multiplying the shear stress by the area (A) of the track segment, as shown in Equation (25):

(25) $$F_{shear}=\tau_{shear}A$$

**Figure 4:**
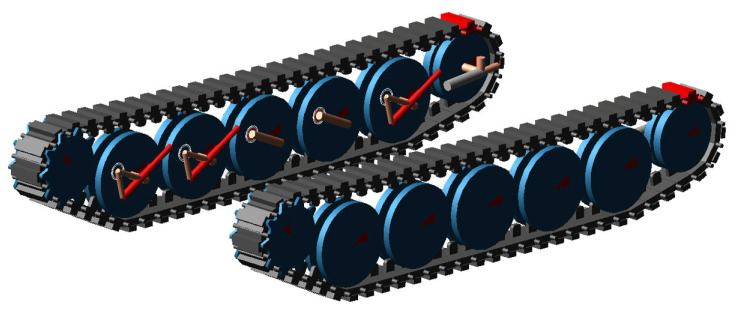
MSC Adams ATV model.

Suspension components, consisting of torsion bars, dampers, sprockets, idlers, track adjusters, rod-arms, and road wheels, were modeled physically (Figure 4).

**Figure 5:**
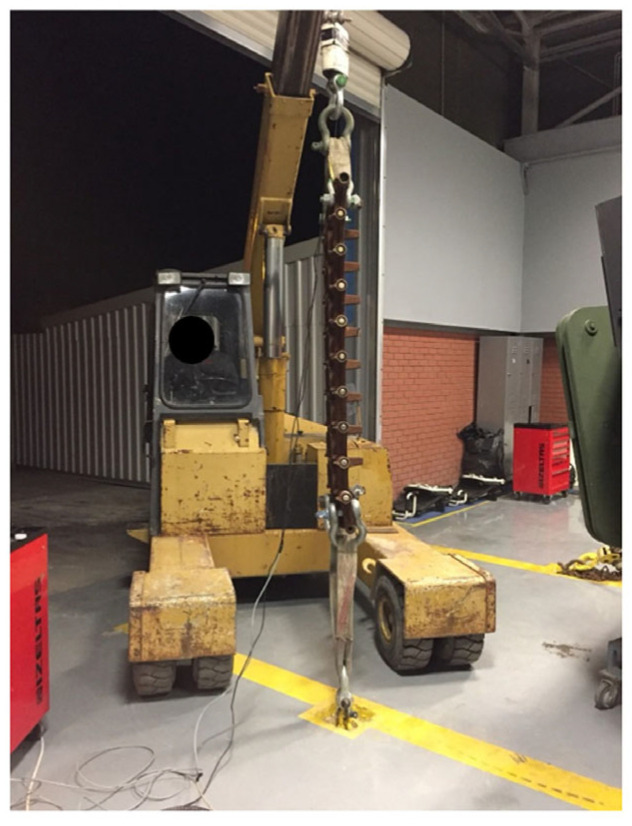
Track longitudinal stiffness test.

Track vertical and longitudinal stiffness were input to the model from bench tests. One example of a pull test, which was conducted with force transducers, is shown in Figure 5.

## 4. Pure Pursuit Algorithm

The pure pursuit algorithm generates the required curvature demand for a robot to converge on a desired path. For a given desired path and the instantaneous position of the vehicle, a goal point, which is at a certain distance from the vehicle, is computed. This distance is called the look-ahead distance because it resembles a point where a driver looks with his/her eyes to direct the vehicle along a curve [6]. Based on the goal point and the cross-track error, right- and left-track velocities are regulated to comply with the required curvature. Since it is a kinematic controller, it does not consider parameters such as vehicle mass, inertia or track forces.

The same look-ahead distance formulation proposed by Wang [11] was used in this study because it is simple to implement and works satisfactorily (Equation (26)):

(26) $$L_{d}=L_{dmin}+k_{1}\;v_{xB}-k_{2}\gamma$$

In Equation (34), the minimum look-ahead distance that can be specified is denoted as $L_{dmin}$, the vehicle’s desired forward velocity in km/h is $v_{xB}$, and the path curvature is denoted as γ. This formulation ensures a smooth response by increasing the look-ahead distance as the vehicle’s forward velocity increases. The look-ahead distance is decreased when the curvature of the reference path increases. If the look-ahead distance is too close, the vehicle exhibits oscillatory behavior, whereas if it is too high, the look-ahead distance causes slow convergence on the desired path [6]. A lower and upper bound for the look-ahead distance is determined via iterative simulations. The velocity-tuning parameter $k_{1}$ was selected such that the resulting distance was within the prescribed bounds covering most of the operational velocity range corresponding to an interval of 3–8 m. The curvature-tuning parameter $k_{2}$ was kept the same as in the study by Wang [11]. The determined $k_{1}$ and $k_{2}$ parameters were used both in the simulation domain and field tests.

### 4.1. Classical Pure Pursuit

**Figure 6:**
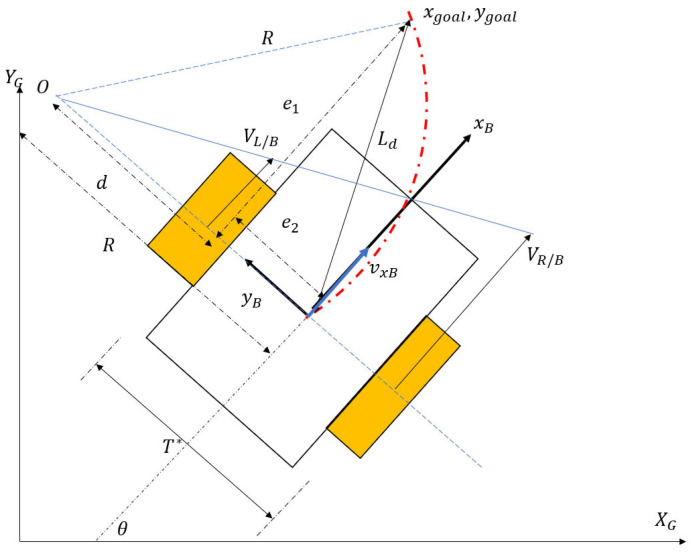
DDR pure pursuit.

For a differential drive robot (DDR) with no slip, the following Equations (27)–(32) can be written by referring to Figure 6.

(27) $$d=R-e_{2}$$

(28) $$(R-e_{2}{)}^{2}+{e_{1}}^{2}=R^{2}$$

(29) $$R^{2}-2Re_{2}+{e_{2}}^{2}+{e_{1}}^{2}=R^{2}$$

(30) $$2Re_{2}=L_{d}^{2}$$

(31) $$R=\frac{L_{d}^{2}}{2e_{2}}$$

(32) $$\gamma =\frac{2e_{2}}{L_{d}^{2}}$$

The required curvature of the path to guide the vehicle to the goal point ($x_{goal},y_{goal})$ is related to the cross-track error and the look-ahead distance in Equation (40). Control inputs, which are the right- and left-wheel angular velocities, can be derived from the simple kinematics in Equations (33) and (34):

(33) $$\omega_{L}=\frac{V_{L/B}}{r_{spr}}=\frac{v_{xB}}{r_{spr}}-\frac{\dot{\theta }*T}{2r_{spr}}=\frac{v_{xB}}{r_{spr}}-\frac{v_{xB}T}{2Rr_{spr}}=\frac{v_{xB}}{r_{spr}}-\frac{v_{xB}T\gamma }{2r_{spr}}=\frac{v_{xB}}{r_{spr}}\left(1-\frac{Te_{2}}{L_{d}^{2}}\right)$$

(34) $$\omega_{R}=\frac{V_{R/B}}{r_{spr}}=\frac{v_{xB}}{r_{spr}}+\frac{\dot{\theta }*T}{2r_{spr}}=\frac{v_{xB}}{r_{spr}}+\frac{v_{xB}T}{2Rr_{spr}}=\frac{v_{xB}}{r_{spr}}+\frac{v_{xB}T\gamma }{2r_{spr}}=\frac{v_{xB}}{r_{spr}}\left(1+\frac{Te_{2}}{L_{d}^{2}}\right)$$

Equations (33) and (34) imply that cruise control and lateral path-tracking problems can be treated separately. For a desired cruise control speed, a corresponding angular velocity is requested by the $\frac{V_{xB}}{r_{w}}$ term. The necessary speed adjustments required for lateral control are taken care of by the $\frac{Te_{2}}{L_{d}^{2}}$ terms, producing a speed differential, which causes the vehicle to steer.

### 4.2. Pure Pursuit with Extended Kalman Filter

**Figure 7:**
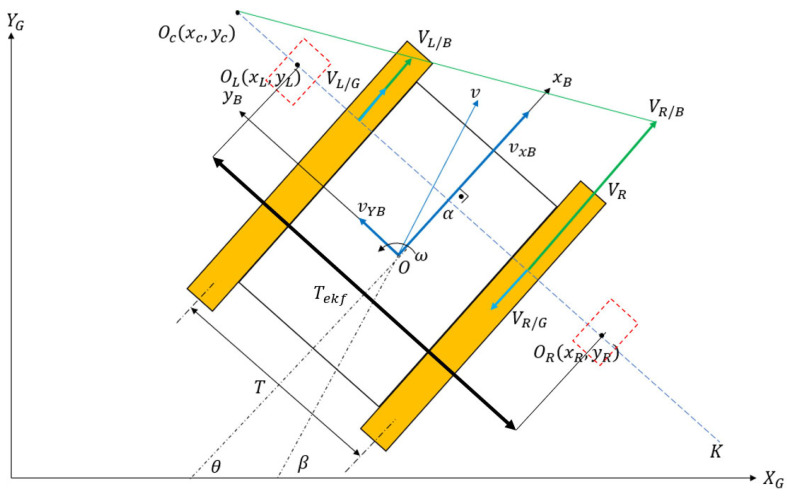
Pure pursuit with slip.

To make the classical pure pursuit ‘slip aware’, the EKF algorithm, which was introduced in Section 2, was incorporated into the algorithm. Referring to Figure 7, it can be observed that the tracked robot behaves like a DDR at the points $O_{L}$ and $O_{R}$ because the vehicle does not have lateral velocity along the $O_{c}-K$ axis.

The velocity requests for this artificial DDR were modified, as in Equations (35) and (36):

(35) $$\omega_{L}=\frac{V_{L/B}}{r_{spr}}=\frac{V_{xB}}{r_{spr}}\left(1-\frac{T_{ekf}e_{2}}{L_{d}^{2}}\right)$$

(36) $$\omega_{R}=\frac{V_{R/B}}{r_{spr}}=\frac{V_{xB}}{r_{spr}}\left(1+\frac{T_{ekf}e_{2}}{L_{d}^{2}}\right)$$

where the artificial track width is computed from Equation (37):

(37) $$T_{ekf}=\left|y_{L}\right|+\left|y_{R}\right|$$

**Figure 8:**
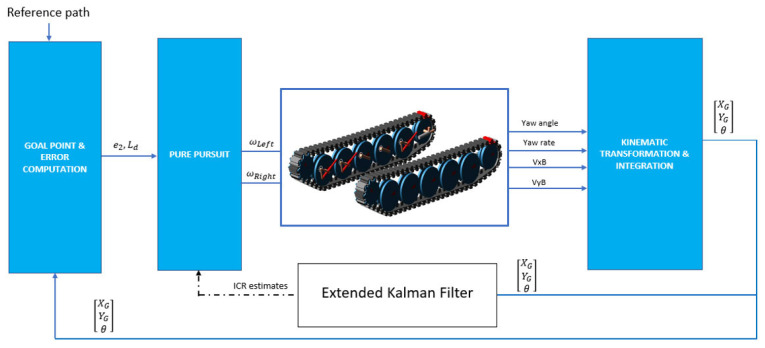
Block diagram of the algorithm.

The EKF equation given in Equation (17) outputs ICR location estimates, which are used by Equations (35) and (36) to regulate the speed demands. The algorithm replaces the constant track width of vehicle T with an artificial track width $T_{ekf}$, which is the summation of the absolute values of $y_{L}$ and $y_{R}$. To demand realistic speed differentials between the right and left sprockets, the upper values of the $\left|y_{L}\right|$ and $\left|y_{R}\right|$ terms were saturated. A block diagram of the proposed architecture is illustrated in Figure 8.

The pure pursuit control algorithm and extended Kalman filter were implemented in the MATLAB–Simulink R2023b (Mathworks, Natick, MA, USA) environment, whereas the tracked platform model was developed in the MSC Adams Tracked Vehicle 2022.1 (ATV) module. MSC Adams and MATLAB–Simulink were co-simulated via the Functional Mockup Interface (FMI) 2.0.

The nomenclature used in this section is presented in Table 3.

## 5. Path-Tracking Simulation Results

The developed high-fidelity model was co-simulated between MATLAB Simulink–MSC Adams for two path-tracking scenarios. The first scenario is a fictitious test track, which is made of flat, semi-circular and lane-change segments. The second scenario is a slalom-type maneuver, for which the vehicle follows a sinusoidal route. The performance of two control algorithms was compared: classical pure pursuit and pure pursuit with the EKF. The classical pure pursuit is designated as T because it has a constant track width T, while pure pursuit with the EKF is designated as $T_{ekf}$ because the speed requests are generated for a variable artificial track width. A common performance measure called lateral error, which is the perpendicular distance between the vehicle’s cog and the two points formed by the closest waypoints, was used to assess the path-tracking performance. The vehicle and simulation parameters are detailed in Table 4.

**Figure 9:**
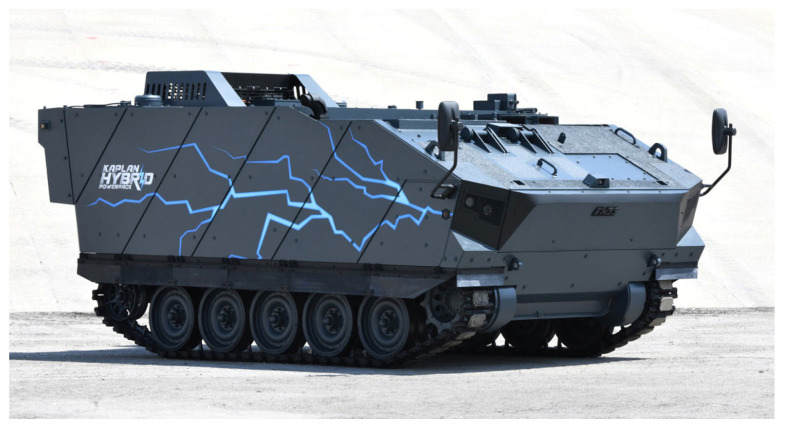
Off-road tracked platform [21].

The simulated vehicle is a tracked off-road vehicle with a weight of 16 tons. It is a prototype series hybrid dual motor robotic platform, which was introduced in a study by Çeliksöz et al. [20], and is shown in Figure 9.

**Figure 10:**
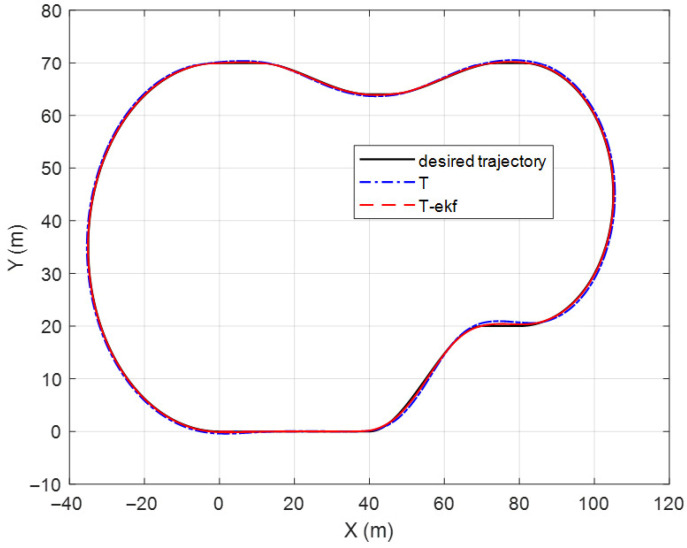
Fictitious test track.

**Figure 11:**
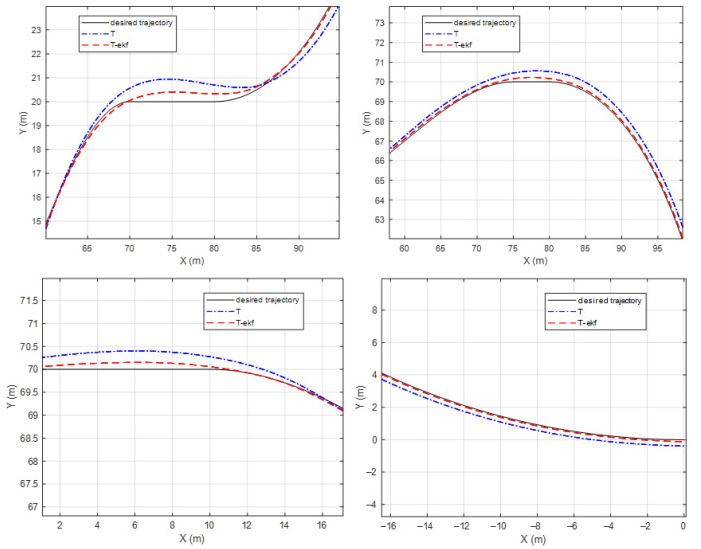
Fictitious test track—zoomed-in segments.

**Figure 12:**
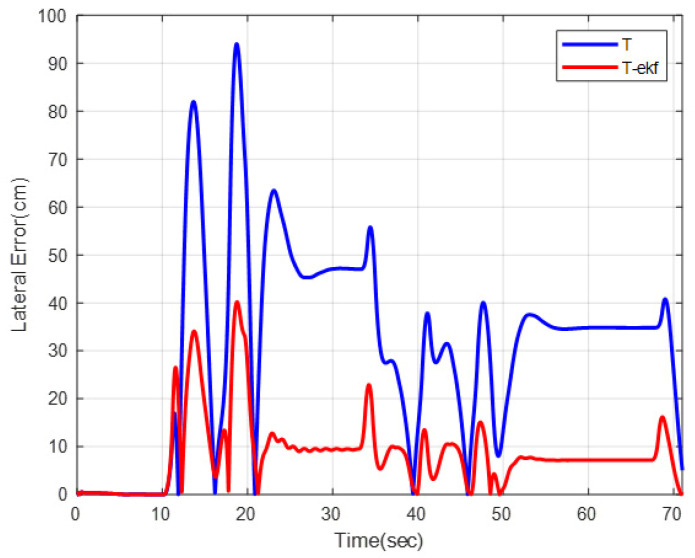
Lateral errors for fictitious test track.

The results of the fictitious test track with a forward velocity of 20 km/h are shown in Figure 10 and Figure 11. The lateral error comparisons for the same route are detailed in Figure 12.

The maximum lateral errors are 94 cm and 36 cm for the classical and EKF algorithms, respectively. The mean absolute errors (maes) are 31 cm and 7 cm, respectively. The estimated ICR locations give the controller a significant advantage by decreasing the amount of overshoot. The zoomed-in sections shown in Figure 11 demonstrate this statement.

A slalom-type simulation scenario was performed to further investigate the performance of the algorithm on a sinusoidal route. The vehicle was expected to track the path detailed in Equation (38), with a cruise velocity of 10 km/h:

(38) $$Y_{G}=5\sin\left(\frac{\pi }{20}X_{G}\right)$$

**Figure 13:**
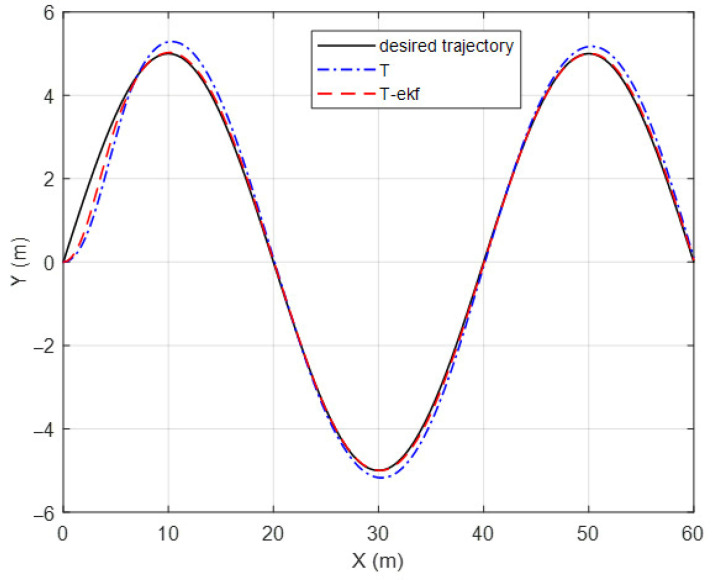
Slalom-maneuver trajectory.

**Figure 14:**
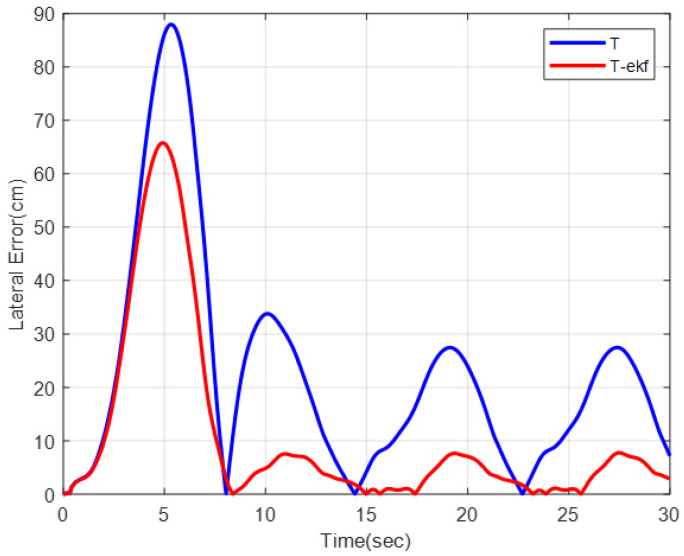
Lateral errors for slalom trajectory.

As can be observed from Figure 13 and Figure 14, the EKF-enhanced pure pursuit has better tracking accuracy in sinusoidal movement as well. A comparison table of the lateral errors is shown in Table 5.

## 6. Field Verifications

This section presents the field verifications. The verifications are grouped into two categories: verification of the high-fidelity model and verification of the proposed control algorithm. The test vehicle was equipped with various sensors, such as SGB (SGB Systems, Rueil-Malmaison, France) Ellipse-D IMU, VBOX 4 (VBOX Automotive, Buckingham, UK) Racelogic GPS, Kvaser (Kvaser AB, Mölndal, Sweden) and shaft encoders. They were connected to a data acquisition system. The test vehicle could be driven by a driver or in an unmanned mode. The vehicle was driven by a driver in the tests presented in Section 6.1, while it was in the unmanned mode in the results presented in Section 6.2.

### 6.1. Dynamic Model Verifications

**Figure 15:**
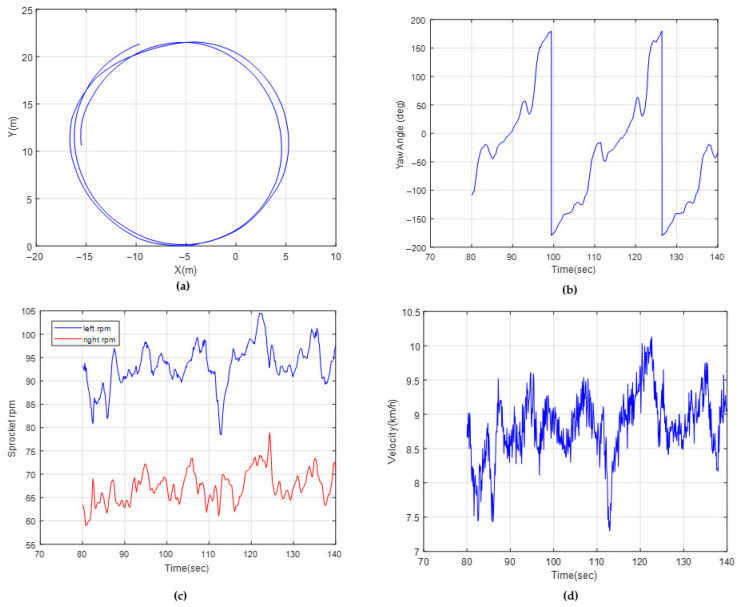
Test data for R 11 m: (**a**) path coordinates, (**b**) yaw angle, (**c**) sprocket rpms, (**d**) vehicle velocity.

**Figure 16:**
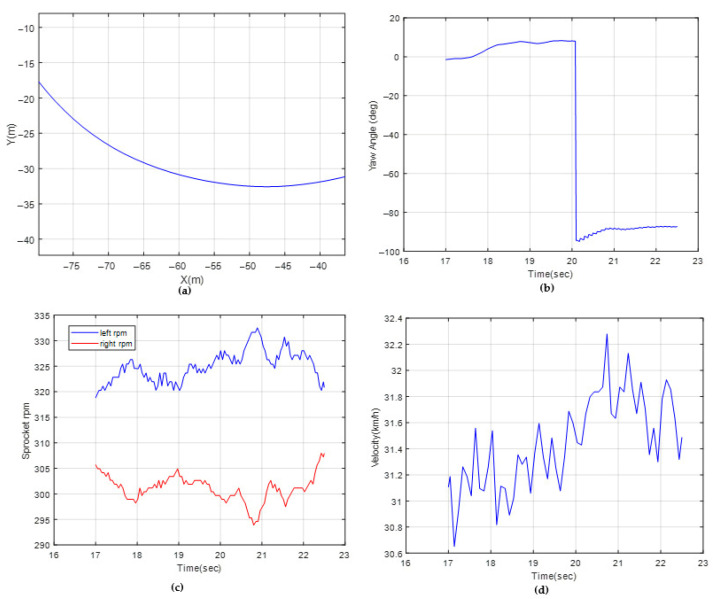
Test data for R 70 m: (**a**) path coordinates, (**b**) yaw angle, (**c**) sprocket rpms, (**d**) vehicle velocity.

Tests were performed on a stabilized dirt road with hard terrain. To sweep different lateral acceleration and velocity profiles, different circular trajectories were created by means of traffic cones. They were used as visual guides to ensure the driver followed the desired path. The driver was instructed to maintain a specified velocity as he followed the predetermined route. The tests were repeated at least two times. The investigated circular trajectories were R 11 m, R 25 m and R 70 m, with velocity profiles of 10 km/h, 15 km/h and 30 km/h, respectively. In the simulations, the vehicle was driven along the same radius circular trajectories. Figure 15 shows the sensor results of a circular trajectory with a radius of 22 m, for which the driver aimed to drive at a velocity of 10 km/h. Figure 16 shows the measurements with a radius of 70 m, for which the driver aimed to drive at a velocity of 30 km/h. Table 6 shows the test and simulation comparisons for three tests in terms of the vehicle yaw rate and sprocket speed difference. It should be noted that the soil parameters shown in Equation (31) (c, ∅ and K) were modified, such that the R11 m results are within +/−2% of the test results. The remaining tests were then compared with the simulations to provide an indication of the accuracy of the dynamic model.

### 6.2. Field Experiments

**Figure 17:**
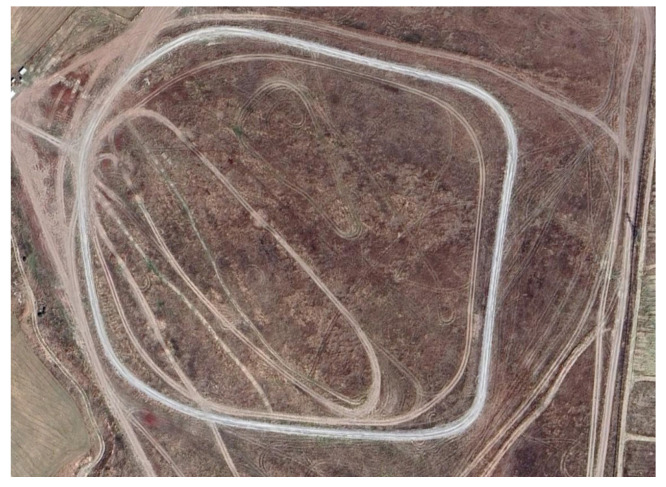
A satellite image of the test parkour.

The test platform was driven along a specified route in the unmanned driving mode, and the vehicle data was recorded. The motion control algorithm is a sub-block of the overall software package. It was embedded into the vehicle ECU by code generation and rapid control prototyping hardware. The vehicle traveled along the outer periphery of the satellite image, as shown in Figure 17.

**Figure 18:**
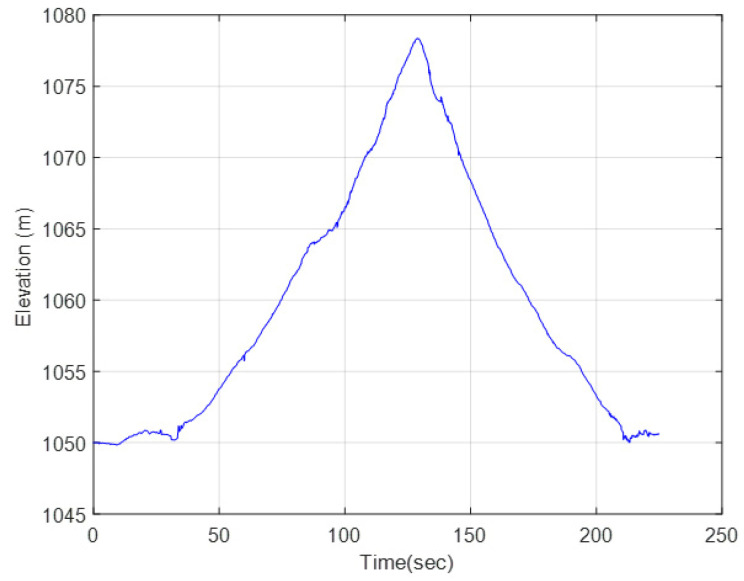
GPS elevation data.

**Figure 19:**
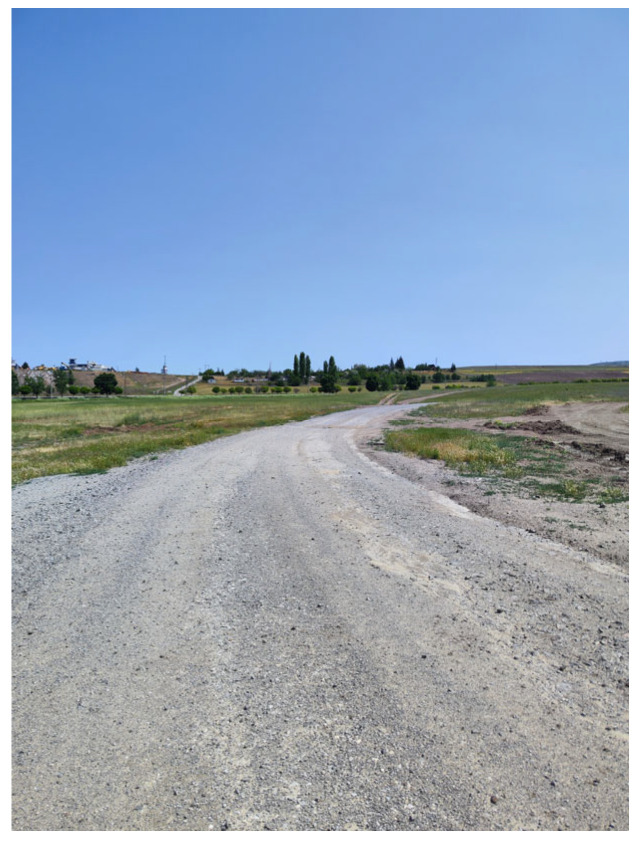
Stabilized dirt road.

As shown in Figure 18, the route is not flat and exhibits elevation, corresponding to roughly a 7% longitudinal grade. The terrain is a hard, stabilized dirt road (Figure 19).

The logged data was compared with the real-time EKF estimations to assess the performance. The process noise covariance matrix **Q** and measurement noise covariance matrix **R** are specified in Equations (39) and (40):

(39) $$Q=\left[\begin{array}{cccccc} 0.1^{2} & 0 & 0 & 0 & 0 & 0\\ 0 & 0.1^{2} & 0 & 0 & 0 & 0\\ 0 & 0 & {\left(\frac{\pi }{180}\right)}^{2} & 0 & 0 & 0\\ 0 & 0 & 0 & 0.05^{2} & 0 & 0\\ 0 & 0 & 0 & 0 & 0.05^{2} & 0\\ 0 & 0 & 0 & 0 & 0 & 0.05^{2} \end{array}\right]$$

(40) $$R=\left[\begin{array}{ccc} 0.03^{2} & 0 & 0\\ 0 & 0.03^{2} & 0\\ 0 & 0 & {\left(\frac{\pi }{180}\right)}^{2} \end{array}\right]$$

Another important factor that affects the filter performance is the selection of the ICR initial conditions. The developed multi-body dynamics model was simulated for a circular path for different radii to sweep different lateral accelerations. In a steady-state circular motion, ICR locations converge to constant values. The tabular results for CW and CCW directions are shown in Table 7.

According to the results, the $y_{L}$ initial condition is given as the average of four trials of CCW runs (1.75 m), while the $y_{R}$ initial condition is given as the average of four trials of CW runs (−1.67 m).

**Figure 20:**
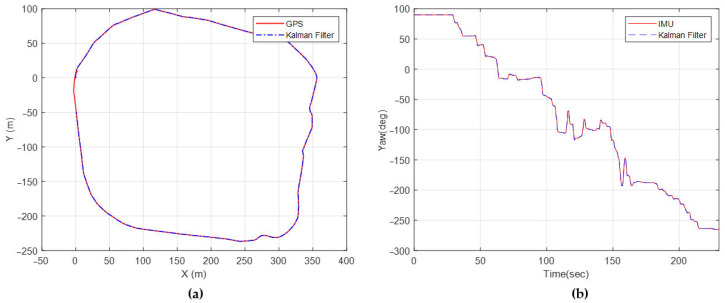
(**a**) Position estimation, (**b**) yaw angle estimation.

A comparison of position estimations and GPS data is shown in Figure 20a. The yaw angles measured by IMU, along with the yaw angle estimations, are shown in Figure 20b. The results are very much in agreement with the test data. This conclusion is similar to the study of Pentzer [15] because the sensors used are quite accurate. Also, the first three states were corrected at each time step with the sensor measurements.

**Figure 21:**
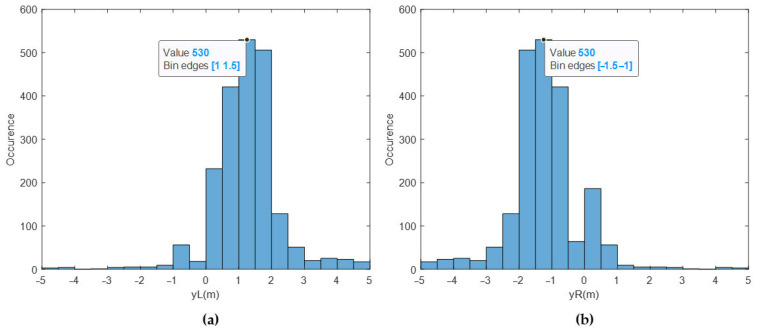
(**a**) Histogram of $y_{L}$ from sensor data, (**b**) histogram of $y_{R}$ from sensor data.

The lateral locations of the ICRs ($y_{L}$ and $y_{R}$), which exhibit scattered behavior, were also extracted from the sensor readings. They were calculated via Equations (7) and (8) at each time step. The histogram values of the sensor readings are shown in Figure 21.

**Figure 22:**
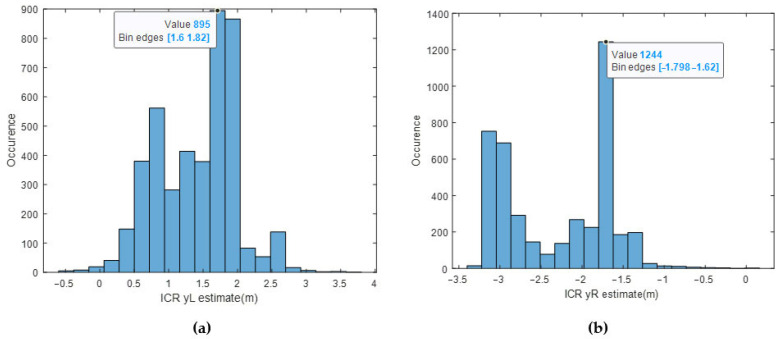
(**a**) Histogram of $y_{L}$ estimate via EKF, (**b**) histogram of $y_{R}$ estimate via EKF.

The histograms of ICR location estimates using the EKF for the same test are presented in Figure 22.

The sensor readings for $y_{L}$ are clustered around 1.5 m, whereas the $y_{L}$ estimates are clustered around 1.7 m. The sensor readings for $y_{R}$ are clustered around −1.5 m, whereas the $y_{R}$ estimates are clustered around −1.75 m. It can be deduced that the designed EKF outputs good enough ICR locations from a statistical point of view, such that the majority of the data is within the predicted bounds [−3 m, 3 m]. In Pentzer’s [15] study, it was concluded that the $y_{L}$ and $y_{R}$ locations are bounded and finite. This study reaches a similar conclusion; however, the ICRs are more scattered, probably due to the weight and dimension difference between the platforms.

Another observation is the summation of the absolute values of $y_{L}$ and $y_{R}$ for the dominant case yields a 3 m value for $T_{ekf}$ while the original track width is 2.2 m. This corresponds to an amplification of 36%, demonstrating the significant deviation of the vehicle from a DDR approach with no slip.

**Figure 23:**
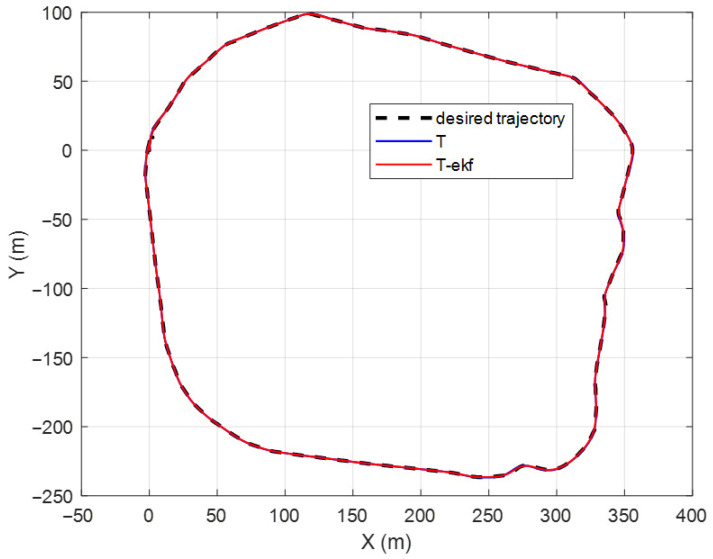
Resulting paths for different control algorithms.

**Figure 24:**
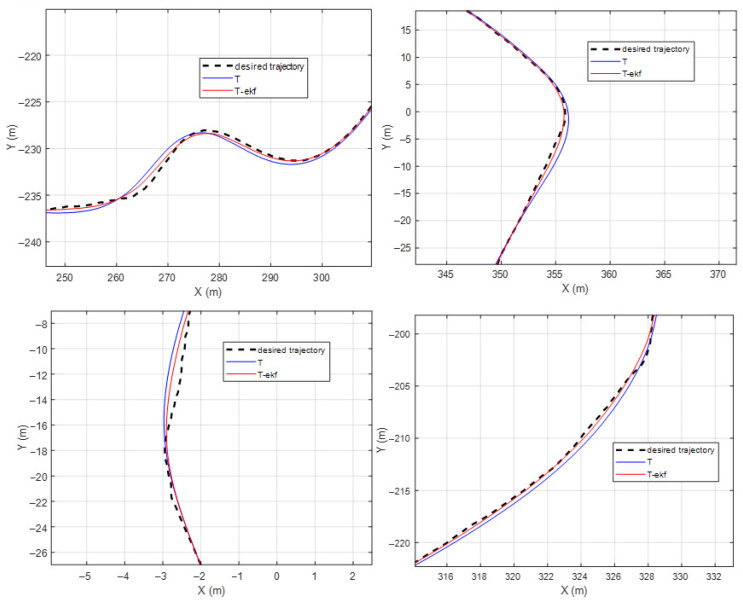
Resulting paths for different control algorithms—zoomed-in segments.

**Figure 25:**
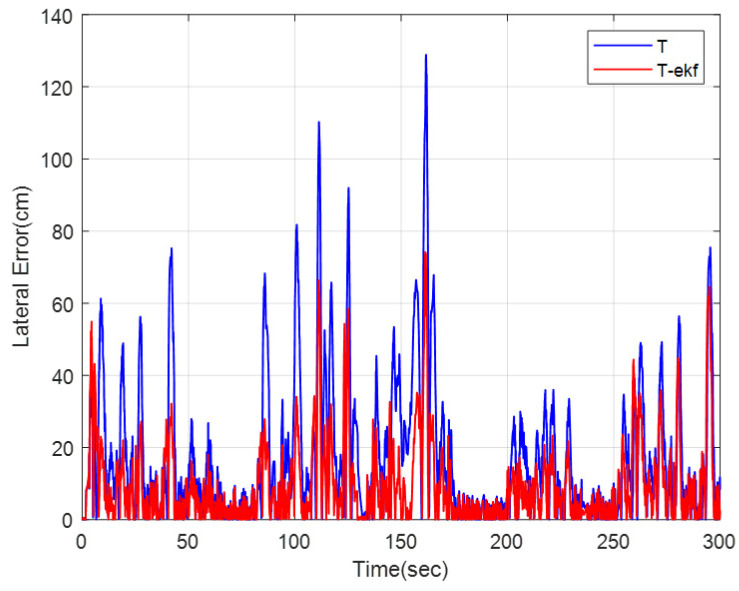
Lateral errors for different control algorithms.

The motion control comparisons of the classical pure pursuit and the EKF-enhanced version on the same parkour are shown in Figure 23 and Figure 24. The residual error for both algorithms is shown in Figure 25.

The EKF-enhanced algorithm outperforms the classical pure pursuit in the field tests as well. The mean absolute errors for the entire route are 17.1 cm and 9.6 cm for the classical and EKF methods, respectively. This corresponds to a 44% decrease in residual errors, which is quite significant.

## 7. Discussion

This study presents a method for improving the pure pursuit algorithm’s path-tracking capability for heavyweight tracked robots. The motivation for the investigation of the pure pursuit algorithm comes from three major reasons. First of all, it has no dependence on system parameters such as road characteristics, vehicle resistances or track–terrain interaction. For an off-road tracked vehicle, these parameters vary a lot, which makes it quite challenging to treat them as known parameters or identify them in real time. Secondly, the simple algebraic relations in the algorithm are easy to code, and they require little computational resources. This avoids purchasing expensive real-time deployment processors. Therefore, for a prototype vehicle, as in the case of this paper, a simple yet accurate enough controller is the ideal choice. Finally, unlike classical kinematic controllers such as PID, the pure pursuit algorithm starts to apply lateral control before entering a curve using the look-ahead distance formulation, which is a significant advantage in heavyweight platforms.

By utilizing an extended Kalman filter, the slip centers of tracks were estimated using onboard sensor measurements from the vehicle. This way, a kinematic algorithm was modified heuristically to consider the slippage effects while avoiding solving complex track–ground interaction equations. This hybrid approach, which regulates the speed difference of the sprockets by means of ICR estimation, is the claimed contribution of this paper. The verification of the compared architectures, both in the simulation environment and field tests, demonstrates the effectiveness of the method. The animations of the multi-body simulations can be accessed from the link given in Supplementary Materials section.

The DDR with no slip approach and the EKF approach were presented for a virtual test track in a co-simulation domain. The maximum errors decrease from 94 cm to 36 cm, while the mean absolute errors decrease from 31 cm to 7 cm in the virtual track with the EKF-enhanced method. In the field tests, the vehicle was driven along a stabilized dirt track. The mean absolute errors were calculated as 17.1 cm to 9.6 cm, which equals an approximate 44% improvement in residual errors.

The minor disadvantages of the EKF methodology compared to the classical pure pursuit should also be pointed out. The filter needs logical initial conditions to converge quickly in real time. The high-fidelity simulation model provides those required initial conditions; however, such a model may not always be available. Furthermore, the process noise covariance matrix was tuned by trial and error, which can be time-consuming in field tests.

Some future research opportunities are listed as follows: The presented ICR estimation method can be used by different kinematic controllers other than pure pursuit, such as backstepping kinematics or the Stanley method, to assess whether similar performance improvements can be achieved. The performance of the state estimator at the moments when the vehicle changes from one terrain to another, such as from mud to sand, with different soil characteristics, can be investigated. The presented results of the paper are valid for a stabilized dirt road with minimal sinkage of the pads into the soil. Currently, track angular velocity requests generated by the algorithm are realized with a first-order transfer function delay. A low-level torque controller that regulates the sprocket speeds can better reflect the actuation dynamics. This torque controller would also handle the transmission output upper/lower torque limits with respect to rpm. The look-ahead distance parameters $k_{1}$ and $k_{2}$ can be determined more elegantly with an online optimization method to reduce the lateral errors even further.

## Figures and Tables

**Table 1:** A comparative table of the methods discussed.

Method	Advantage	Limitation/Application
Classical Pure Pursuit [10,11,12,13]	Easy to implement Real-time cost efficiency Look-ahead nature	Disregards slip Limited to low speeds Path-tracking application
Kinematics-Aware MPC Zhao [16]	Optimal control by considering future states, considers slip, accurate path tracking up to 30 km/h	High computational cost because of real-time optimization, path-tracking application
EKF Pentzer [15]	Provides a method to estimate ICR locations in real time	Dead-reckoning navigation application
Pure Pursuit + EKF	Easy to implement, computationally efficient, more accurate than classical pure pursuit	Not as accurate as MPC at high speeds Path-tracking application

**Table 2:** The nomenclature presented in the section.

Parameter	Description
$V_{R/G},V_{L/G}$	Right-/left-track velocity with respect to ground (m/s)
$V_{R/B},V_{L/B}$	Right-/left-track velocity with respect to vehicle body (m/s)
θ ,ω ,β	Vehicle yaw angle, yaw rate, heading angle (rad)
$X_{G},Y_{G}$	Global coordinates in inertial frame (m)
$v_{xB},v_{yB}$	Longitudinal and lateral velocities in body frame (m/s)
$y_{R},y_{L},y_{c}$	Lateral position of ICRs in body frame with respect to point O (m)
$x_{R},x_{L},x_{c}$	Longitudinal position of ICRs in body frame with respect to point O (m)
T	Vehicle track width (m)
$\omega_{L},\omega_{R}$	Angular velocity of right/left tracks (rad/s)
$r_{spr}$	Sprocket pitch radius (m)
$x_{k}$	State vector at the kth time step
$u_{k}$	Input vector at the kth time step

**Table 3:** Descriptions of the nomenclature presented in Section 4.

Parameter	Description
$L_{d}$	Look-ahead distance (m)
$e_{1},\;e_{2}$	Along-track error/cross-track error (m)
$k_{1},\;k_{2}$	Velocity-tuning parameter/curvature-tuning parameter
R	Instantaneous turning radius (m)
$x_{goal},y_{goal}$	Goal coordinates in global frame (m)
γ	Instantaneous curvature (1/m)
$T_{ekf}$	Artificial track width estimated by EKF (m)

**Table 4:** Vehicle and simulation parameters.

Parameter	Value
Combat weight	16,000 (kg)
Number of roadwheels per side	5
Yaw inertia	22,325 (kg m^2^)
Height of cog	1.03 (m)
Track width	2.17 (m)
Length of track contact	2.67 (m)
Peak friction coefficient	0.9
L_dmin_	3 (m)
k_1_–k_2_	0.24–0.1
MATLAB–Simulink Solver	Runge Kutta ode4
MSC Adams Solver	HHT
Fixed step size	0.025 s

**Table 5:** Lateral error comparisons.

	Classical PP Maximum Error (cm)	EKF + PP Maximum Error (cm)	Classical PP Mae (cm)	EKF + PP Mae (cm)
Fictitious track	94	36	31	7
Slalom	88	66	23	11

**Table 6:** Test–simulation comparisons.

	Test (Mean)	Simulation	Percent Error
Yaw velocity (deg/s)—R 11 m	13.1	13.4	2%
Sprocket speed difference (rpm)—R 11 m	32.4	33.1	2%
Yaw velocity (deg/s)—R 25 m	9.9	9.1	−8%
Sprocket speed difference (rpm)—R 25 m	20.7	22.3	8%
Yaw velocity (deg/s)—R 70 m	8.2	7.5	−9%
Sprocket speed difference (rpm)—R70 m	19.7	21.1	7%

**Table 7:** ICR locations for circular trajectory.

Lat Acc (g)	$y_{L}$ (m)	$y_{R}$ (m)	$y_{C}$ (m)	$x_{L}$ (m)
0.1-CCW	1.70	−1.48	70.11	0.19
0.2-CCW	1.63	−1.47	36.55	0.37
0.3-CCW	1.73	−1.39	24.64	0.68
0.4-CCW	1.92	−1.33	18.59	0.84
0.1-CW	1.41	−1.75	−70.31	0.19
0.2-CW	1.26	−1.65	−34.90	0.40
0.3-CW	1.35	−1.64	−24.90	0.64
0.4-CW	1.30	−1.66	−19.22	0.79

## Data Availability

The data presented in this study are available on request from the corresponding author. The data are not publicly available due to privacy restrictions.

## Supplementary Materials

Videos of the simulations can be accessed from the following links: https://www.youtube.com/watch?v=LvjMykoOOWc (accessed on 30 June 2025). https://www.youtube.com/watch?v=4LV--hdwTWE (accessed on 30 June 2025).

## Abbreviations

The following abbreviations are used in this manuscript:

DDR	Differential Drive Robot
EKF	Extended Kalman Filter
UGV	Unmanned Ground Vehicle
ICR	Instantaneous center of rotation
FMI	Functional Mockup Interface
MAE	Mean Absolute Error

## Appendix A

The equations shown in this appendix are for the calculation of Jacobian matrices and the propagation of the state estimates presented in Section 2:

(A1) $$P_{k}^{-}=F_{k-1}P_{k}^{+}F_{k-1}^{T}+L_{k-1}Q_{k-1}L_{k-1}^{T}$$

(A2) $$F_{k-1}={\left.\frac{\partial f_{k-1}}{\partial x}\right|}_{x_{k-1}^{+}}$$

(A3) $$L_{k-1}={\left.\frac{\partial f_{k-1}}{\partial w}\right|}_{x_{k-1}^{+}}=∆t\;I_{6x6}$$

(A4) $$h_{k}=\left[\begin{array}{c} X_{Gk}+{MN}_{1}\\ Y_{Gk}+{MN}_{2}\\ \theta_{k}+{MN}_{3} \end{array}\right]$$

(A5) $$H_{k}={\left.\frac{\partial h_{k}}{\partial x}\right|}_{x_{k}^{-}}=\left[\begin{array}{ccc} 1 & 0 & 0\\ 0 & 1 & 0\\ 0 & 0 & 1 \end{array}\;\;\begin{array}{ccc} \;0 & 0 & 0\\ \;0 & 0 & 0\\ \;0 & 0 & 0 \end{array}\right]$$

(A6) $$M_{k}={\left.\frac{\partial h_{k}}{\partial v}\right|}_{x_{k}^{+}}=I_{3x3}$$

(A7) $$K_{k}=P_{k}^{-}H_{k}^{T}{\left(H_{k}P_{k}^{-}H_{k}^{T}+M_{k}R_{k}M_{k}^{T}\right)}^{-1}$$

(A8) $$x_{k}^{+}=x_{k}^{-}+K_{k}\left[y_{k}-h_{k}\left(x_{k}^{-},0\right)\right]$$

(A9) $$P_{k}^{+}=\left(I_{6x6}-K_{k}H_{k}\right)P_{k}^{-}$$

**Table A1:** Descriptions of the nomenclature.

Parameter	Description
$P_{k}^{-}$	A priori state covariance matrix at the kth step
$F_{k},L_{k},H_{k},M_{k}$	Jacobian matrices
$K_{k}$	Kalman gain
$x_{k}^{+}$	A posteriori state estimate
$P_{k}^{+}$	A posteriori state covariance matrix at the kth step
